# GK-1 Induces Oxidative Stress, Mitochondrial Dysfunction, Decreased Membrane Potential, and Impaired Autophagy Flux in a Mouse Model of Breast Cancer

**DOI:** 10.3390/antiox12010056

**Published:** 2022-12-27

**Authors:** Alfredo Cruz-Gregorio, Ana Karina Aranda-Rivera, Omar Emiliano Aparicio-Trejo, Omar Noel Medina-Campos, Edda Sciutto, Gladis Fragoso, José Pedraza-Chaverri

**Affiliations:** 1 Laboratorio F-315, Departamento de Biología, Facultad de Química, Universidad Nacional Autónoma de México (UNAM), Mexico City 04510, Mexico; 2 Departmento de Fisiopatología Cardio-Renal, Instituto Nacional de Cardiología “Ignacio Chávez”, Mexico City 14080, Mexico; 3 Departamento de Inmunología, Instituto de Investigaciones Biomédicas, Universidad Nacional Autónoma de México (UNAM), Mexico City 04510, Mexico

**Keywords:** GK-1, oxidative stress, VDAC, ATP synthase, mitochondrial dysfunction, autophagy flux

## Abstract

Breast cancer (BC) is the second most common cancer worldwide in women. During the last decades, the mortality due to breast cancer has progressively decreased due to early diagnosis and the emergence of more effective new treatments. However, human epidermal growth factor receptor 2 (HER2) and triple-negative breast cancer (TNBC) remain with poor prognoses. In our research group, we are proposing the GK-1 immunomodulatory peptide as a new alternative for immunotherapy of these aggressive tumors. GK-1 reduced the growth rate of established tumors and effectively reduced lung metastasis in the 4T1 experimental murine model of breast cancer. Herein, the effect of GK-1 on the redox state, mitochondrial metabolism, and autophagy of triple-negative tumors that can be linked to cancer evolution was studied. GK-1 decreased catalase activity, reduced glutathione (GSH) content and GSH/oxidized glutathione (GSSG) ratio while increased hydrogen peroxide (H_2_O_2_) production, GSSG, and protein carbonyl content, inducing oxidative stress (OS) in tumoral tissues. This imbalance between reactive oxygen species (ROS) and antioxidants was related to mitochondrial dysfunction and uncoupling, characterized by reduced mitochondrial respiratory parameters and dissipation of mitochondrial membrane potential (ΔΨm), respectively. Furthermore, GK-1 likely affected autophagy flux, confirmed by elevated levels of p62, a marker of autophagy flux. Overall, the induction of OS, dysfunction, and uncoupling of the mitochondria and the reduction of autophagy could be molecular mechanisms that underlie the reduction of the 4T1 breast cancer induced by GK-1.

## 1. Introduction

Breast cancer (BC) is the most common cancer worldwide in women [1]. BC is highly heterogeneous, with different molecular alterations and clinical behavior differences [2]. This heterogeneity is associated with the expression of different immunohistochemical markers and categorized into four main molecular subtypes of BC tumors: luminal A, luminal B, human epidermal growth factor receptor 2 (HER2)-enriched, and basal-like also known as triple-negative (TNBC). For instance, luminal A and B tumors express the immunochemical markers estrogen receptor (ER) and progesterone receptor (PR) but do not express HER2. These tumors are low-grade, grow slowly, and have the best prognosis. HER2-enriched tumors express HER2 receptors but do not express ER and PR receptors. Regarding TNBC tumors, they do not express ER, PR, and HER2 receptors and are the most lethal and aggressive among BC. Because these tumors do not benefit from hormone therapy or antitumor drugs that target ER, PR, or HER2 protein receptors, there is a need to find alternative targeted therapies for TNBC patients [3]. The use of immunomodulatory peptides offers new strategies for these target therapies [4]. GK-1 is an immunomodulatory peptide derived from the helminth *Taenia crassiceps* that has exhibited antitumor properties in a murine model of breast tumors induced by the 4T1 cell line [5]. 4T1 tumor mimics human breast cancer stage IV, associated with metastasis to the lungs [6,7]. We have shown that treatment with GK-1 significantly lessened the size of the primary tumor, discretely increasing the tumor-bearing mice’s survival. Moreover, GK-1 treatment decreased lung macrometastases, increasing the number of mice free of macrometastases [5]. We have demonstrated that GK-1 exerts its antitumor effect activating the immunomodulatory system [5,8,9,10].

As several antitumoral treatments exert their function through changes in the redox state, mitochondrial metabolism, and autophagy process of the tumoral cells, herein, we explored the effects of GK1 on these systems. The changes in reactive oxygen species (ROS) levels are highly related to cancer development. Meanwhile, some types of cancer require certain ROS levels to grow and develop metastasis; other types of cancer need reduced ROS levels to induce metastasis. For instance, pancreatic cancer cells indeed need ROS to induce metastasis; meanwhile, lung cancer or melanoma are sensitive to ROS, and in OS conditions, cancer and metastasis decrease [11,12,13]. Regarding TNBC, it has been shown that cells have induced high ROS production, where manganese superoxide dismutase (MnSOD) functions as a key pro-oxidant peroxidase that increases mitochondrial ROS (mROS), inducing OS as an oncogenic effect. Interestingly, MnSOD inhibition reduces TNBC progression [14], probably because mROS levels are reduced, and consequently, OS and its oncogenic effect disappear. However, high ROS levels also induce cell death as ROS can overcome the cell tumor umbral of resistance to OS [15]. Thus, TNBC progression depends on mROS but a ROS overproduction could be a therapeutic target to induce TNBC cell death. One mechanism associated with mitochondria is that OS can induce mitochondrial dysfunction and, consequently, cell death [16]. Note that mitochondrial dysfunction also induces ROS in a process that results in positive feedback for ROS overproduction, resulting in tumor cell death [17]. In fact, ROS can induce damage in the electron transport system (ETS), decreasing adenosine triphosphate (ATP) production and increasing mROS production [17]. Since cell death processes are highly regulated by the OS and mitochondria dysfunction, it is also necessary to evaluate the participation of this organelle in the effect of GK-1. Autophagy, and more specifically mitophagy, is a degradation process that can degrade these dysfunctional mitochondria to promote new mitochondria biogenesis [18]. This cellular mechanism eliminates mitochondrial damage, and therefore, whether autophagy is not functioning properly, the tumor decreases because of the accumulation of damaged organelles [19]. Since ROS production, mitochondrial dysfunction, and autophagy are crucial events for cancer and metastasis reduction, we hypothesized that GK-1-induced metastasis and cancer reduction involves mechanisms related to OS and mitochondrial dysfunction, as well as alterations in the autophagy process. To our knowledge, there are no works that have studied the effect of GK-1 in the latter processes. Thus, the aim of this work was to determine the effect of GK-1 on the redox state, mitochondrial metabolism, and autophagy of triple-negative tumors. We demonstrate that the antitumoral effect of GK-1 could also be associated with OS induction, mitochondrial dysfunction, and probably impaired autophagy flux. These findings allow us to better understand the pathways by which GK-1 reduces tumor growth and metastasis, pointing to new co-treatments, that is, GK-1 and other compounds that target the redox state, mitochondria, or cell death by autophagy.

## 2. Materials and Methods

### 2.1. Chemicals

GK-1 was purchased as a synthetic 18-aminoacid peptide (GYYYPSDPNTFYAPPYSA) from USV, LTD, Mumbai, Maharashtra, India (batch RD0001). Antimycin, sodium L-ascorbate, acrylamide, sodium azide (NaN_3_), 5,5-dithio-bis (2 nitrobenzoic) acid (DTNB), 5-thio-2-nitrobenzoic acid (TNB), GSH, GSSG, glutathione reductase (GR), tetramethyl-p-phenylene diamine (TMPD), ethylene glycol-bis(2-aminoethylether)-N,N,N′,N′-tetraacetic acid (EGTA), K-lactobionate, sodium fluoride (NaF), diphenylene iodonium (DPI), diphenyleneiodonium chloride, N-acetyl-l-cysteine (NAC), palmitoyl-carnitine-CoA (PC-C), horseradish peroxidase (HRP), nitroblue tetrazolium (NBT), 4-(2-hydroxyethyl)-1-piperazineethanesulfonic acid (HEPES), nicotinamide adenine dinucleotide phosphate (NADPH), NADP^+^, 1-Chloro-2,4-Dinitrobenzene (CDNB) nicotinamide adenine dinucleotide (NADH), rotenone, phenylmethylsulfonyl fluoride (PMSF), adenosine phosphate (ADP), sodium succinate dibasic, sodium glutamate, sodium phosphate dibasic (Na_2_HPO_4_), sodium phosphate monobasic (NaH_2_PO_4_), potassium phosphate monobasic (KH_2_PO_4_), magnesium chloride (MgCl_2_), sodium dodecyl sulfate (SDS), oligomycin, bovine serum albumin (BSA), sodium orthovanadate (Na_3_VO_4_), xanthine, Tris-HCl, carbonyl cyanide 3-chlorophenylhydrazone (CCCP), triethanolamine, trypan blue, xanthine oxidase, safranin (S), and 2-vinylpyridine (2-VP), were purchased from Sigma-Aldrich (St. Louis, MO, USA). Ethanol, hydrochloric acid, ethylacetate, trichloroacetic acid, hydrogen peroxide (H_2_O_2_), sodium chloride (NaCl), 2,4-dinitrophenylhydrazine (DNPH), and ethylenediaminetetraacetic acid disodium salt dihydrate (EDTA) were obtained from JT Baker (Xalostoc, Edo. Mexico, Mexico). The protease inhibitor cocktail was purchased from Roche Applied Science (Mannheim, Germany). Sevoflurane, USP^®^, was purchased from Baxter, Glenview, IL, USA. The sterile saline solution (SS) was purchased from PiSA Pharmaceutica (Mexico City, Mexico). The rabbit anti-catalase mAb (D4P7B) and rabbit anti-p62 mAb (P00671) were purchased from Cell Signaling (Danvers, MA, USA). Mouse anti-voltage dependent anion channel 1 mAb (VDAC1, sc-390996) was purchased from Santa Cruz Biotechnologies (Dallas, TX, USA). Mouse anti-Beclin mAb (MAB5295) was purchased from R&D Systems (Minneapolis, MN, USA). Mouse anti-glyceraldehyde 3-phosphate dehydrogenase mAb (GAPDH, ab181602) and mouse anti-oxidative phosphorylation (OXPHOS) cocktail mAbs (ab110413) were purchased from Abcam (Cambridge, UK). Secondary antibodies (donkey anti-mouse 680RD, 926-68073; donkey anti-rabbit 800RD, 926-32212; donkey anti-rabbit 800CW, 926-32213) were purchased from LI-COR Biosciences (Lincoln, NE, USA).

### 2.2. Xenotransplantation of 4T1 Cells in BALB/c Mice

Xenotransplantation was performed as reported by Pulaski and Ostrand-Rosenberg [20]. Briefly, viable 4T1 cells were counted by trypan blue dye exclusion to resuspend a total of 1000 cells in 100 µL of SS. The cell suspension was injected subcutaneously into the fat pad of the right breast. All animals were monitored at least every other day to assess tumor growth, which was calculated according to the following formula [21]:

Volume (mm^3^) = π LW^2^/6,

where:

L is the longest side, and W is the shortest side of the tumor.

Once the mice developed palpable tumors (1 mm to 1 mm), they were randomly assigned into two groups (one corresponding to the control group and another group that was treated with GK-1). A total of five independent experiments were performed with 5 mice per group.

### 2.3. Experimental Design

**Figure 1:**
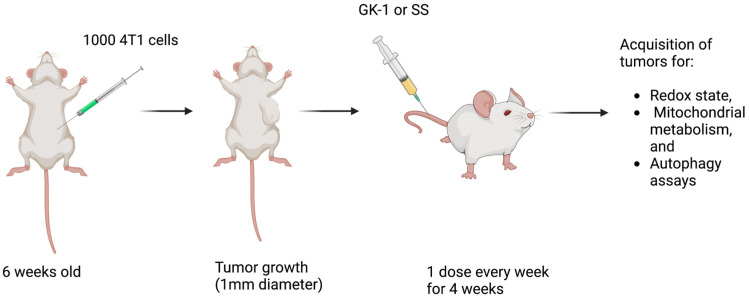
**Experimental design**. Female BALB/c mice were divided into two groups: (1) Control group (treated with saline solution, SS) and (2) GK-1 group, treated with GK-1. SS or GK-1 was administered each week for 4 weeks. At the end of 4 weeks, mice were anesthetized and sacrificed to remove the tumor for analysis. The number of mice for each experiment was 5 per group (n = 5). The figure was created with BioRender.com.

The “Comité Interno para el Cuidado y Uso de los Animales de Laboratorio (CICUAL)” approved the experimental protocol at the “Instituto de Investigaciones Biomédicas of the Universidad Nacional Autónoma de México” (protocol number approval ID 6320). The research was performed according to the Mexican Official Norm Guides for producing, using, and caring for laboratory animals (NOM-062-ZOO-1999). female BALB/c mice were used with an initial age of 6 weeks, divided into two groups after tumor development: (1) the control group treated with SS, and (2) GK-1 treated group. SS or GK-1 were administered each week for 4 weeks. GK-1 was intravenously (i.v.) administered at a 100 mg/100 μL dose (Figure 1). We selected the GK-1 dose according to our previous studies reported in which GK-1 decreases tumors in experimental animal models [5]. The peptide was 95% pure as determined by high-pressure liquid chromatography using a reverse-phase C18 column [22]. The mice were maintained in a temperature-controlled environment with a 12–12 h light–dark cycle, with water and food provided ad libitum. The disposal of biological residues was conducted according to NOM-087-SEMARNAT-SSA1-2002. No animal died during tumor growth or treatment administration. At the end of week 4, the mice were euthanized with sevoflurane excess inhalation, and the tumor was extracted for the analysis.

### 2.4. Antioxidant Enzyme Activity

The antioxidant enzyme activities were evaluated, as previously described by Aparicio-Trejo et al. [23]. Tumor tissues were homogenized (1:5, *w*:*v*) in cold 50 mM potassium phosphate buffer, pH 7.0, containing 0.05% Triton X100. After centrifugation at 15,000× *g* for 30 min at 4 °C, supernatants were obtained for analysis of the following enzymatic assays.

Total superoxide dismutase (SOD) activity was assayed using a superoxide generator system that contained 0.122 mM EDTA, 30.6 μM NBT, 0.122 mM xanthine, 0.006% BSA, and 49 mM sodium carbonate. In a 96-well microplate, 20 μL of the sample was mixed with 0.3 mL of the mixture described above, and then 20 μL of xanthine oxidase (0.1 units (U)/L) was added. Optical density (OD) at 560 nm was read until the delta OD of wells containing H_2_O instead of samples was 0.2 (maximum NBT reduction). The amount of protein in samples that inhibited maximum NBT reduction to 50% was defined as one U of SOD activity. Results were expressed as U/mg protein.

Catalase (CAT) activity was assayed by a method based on the disappearance of H_2_O_2_. Briefly, in a 96-well ultraviolet (UV) microplate, 12 μL of samples were mixed with 340 μL of 30 mmol/L H_2_O_2_, and the OD at 240 nm was measured at 0, 15, and 30 s. The decomposition of H_2_O_2_ by CAT present in the samples follows first-order kinetics according to the equation:

k = 2.3 t log Ao/A

where k is the first-order reaction rate constant, t is the time over which the decrease of H_2_O_2_ due to enzyme activity was measured (15 s), and Ao/A is the ratio of ODs at times 0 to 15 s and 15 s and 30 s, respectively. The results are given in k/mg protein [24].

Glutathione peroxidase (GPx) activity was evaluated, as previously reported by Lawrence and Burk [25], through the disappearance of NADPH at 340 nm in a coupled reaction with GR. Briefly, in a 96-well microplate, 35 μL of samples were added to 280 μL of mixture reaction (1 mM EDTA, 1 mM NaN_3_, 0.2 mM NADPH, 1 U/mL of the GR and 1 mM GSH in 50 mM potassium phosphate, pH 7.0). The reaction was started by the addition of 35 μL of 2.5 mM H_2_O_2_. OD at 340 nm was recorded for 3 min, and the activity was calculated from the slope of these lines using the extinction coefficient of NADPH at 340 nm (6.22 L mmol^−1^cm^−1^). GPx units (U) were defined as the amount of enzyme that oxidizes 1 μmol NADPH/min, and data were expressed as U/mg total protein.

GR activity was evaluated by measuring the disappearance of NADPH at 340 nm. The reaction mixture consisted of 1.25 mM oxidized glutathione, 0.1 mM NADPH, and 0.5 mM EDTA in 100 mM potassium phosphate buffer (pH 7.6). A measure of 17 μL of the sample was mixed with 330 μL of the reaction mixture, and OD at 340 nm was recorded for 3 min and the activity was calculated from the slope of these lines as μmol NADPH oxidized per minute. Here, 1 U of GR was defined as the amount of enzyme that oxidizes 1 μmol NADPH/min. Data are expressed as units per milligram of protein.

Glutathione S-transferase (GST) activity was determined, as previously reported by Habig and Jakoby [26]. The reaction mixture consisted of 2 mM GSH, and 1 mM CDNB in 50 mM potassium phosphate buffer (pH 6.5). In a 96-well microplate, 7 µL of samples were added to 344 µL of the reaction mixture, and the changes in the absorbance due to GSH-CDNB conjugate formation were recorded at 340 nm for 3 min. Enzyme activity was calculated as µmol CDNB conjugate formed/min/mg protein using a molar extinction coefficient of 9.6 × 103/M/cm. Here, 1 U GST was defined as the amount of enzyme that conjugates 1 nmol of CDNB with GSH per minute; data are expressed as U/mg total protein.

### 2.5. Western Blot Analysis

The protein extracts were obtained by adding radioimmunoprecipitation assay (RIPA) lysis buffer (40 mM Tris-HCl, 150 mM NaCl, 2 mM EDTA, 1 mM EGTA, 5 mM NaF, 1 mM Na_3_VO_4_, 1 mM PMSF, 0.5% sodium deoxycholate, 0.1% SDS pH 7.6, supplemented with protease inhibitor cocktail). A total of 20–40 μg of protein was denatured by dilution with 6xx Laemmli sample buffer (60 mM Tris-Cl, pH = 6.8, 2% SDS, 10% glycerol, 5% 2-mercaptoethanol, 0.01% bromophenol blue) and boiled for 5 min. Samples were loaded in SDS-polyacrylamide gels and submitted to electrophoresis. Proteins were transferred to polyvinylidene fluoride membranes (PVDF) and blocked with 5% non-fat dry milk in 0.1% tween-tris buffered solution (TBST) for 1 h at room temperature. Membranes were incubated with the recommended dilutions of antibodies overnight at 4 °C at constant stirring and the corresponding fluorescent secondary antibody (1: 15,000) for 90 min at room temperature in darkness. Primary antibodies were prepared in TBST buffer in the following concentrations: anti-catalase 1:1000 dilution; anti-VDAC1 1:2000 dilution; anti-p62 1:3000 dilution, and anti-Beclin 1:3000 dilution. Anti-GAPDH 1:1000 dilution was used as a loading control. Secondary antibodies were used with a 1:15,000 dilution. The protein bands were detected using fluorescence in an Odyssey Sa Scanner (LI-COR Biosciences, Lincoln, NE, USA). Protein band density was analyzed with ImageJ studio ver.1.48h3, National Institutes of Health (NIH, Bethesda, MD, USA). Quantification of proteins was expressed as arbitrary units, representing the ratio of optical densities of protein of interest/loading control.

#### OXPHOS Protein Determination

The levels of OXPHOS proteins were evaluated by Western blot using an antibody cocktail. This cocktail is composed of a mixture of five antibodies, one against each CI–CIV complex and ATP synthase. These proteins include NADH: ubiquinone oxidoreductase subunit B8 (NDUFB8) for CI (CI-NDUFB8), succinate dehydrogenase B (SDHB) subunit for CII (CII-SDHB), ubiquinol-cytochrome c reductase core protein 2 (UQCRC2) subunit for CIII (CIII-UQCRC2), cytochrome c oxidase I (MTCO1) subunit for CIV (CIV-MTCO1), and ATP5A subunit for ATP synthase. These proteins are the most labile for each complex, and their decrease or increase is related to an alteration in ETS and OXPHOS proteins [27,28]. According to the manufacturer’s instructions, sample proteins were not heated to avoid signal decrease. The membranes were washed three times with phosphate buffer saline/tween 20 (PBST) and then incubated with secondary antibodies. Secondary antibodies were used at a 1:15,000 dilution. The protein bands were detected using fluorescence in an Odyssey Scanner. Densitometric analysis was performed using the ImageJ program.

### 2.6. Isolation of Mitochondria

After sacrifice, tumor tissues were cooled by immersion in isolation buffer (225 mM D-mannitol, 75 mM sucrose, 1 mM EDTA, 5 mM HEPES, 0.1% BSA, pH = 7.4) at 4 °C and then cut into small pieces. Mitochondria were isolated from the tissues mass; tissues were homogenized in a glass Potter–Elvehjem with a TeflonVR pestle in 2 mL of the same buffer. Mitochondria were obtained by differential centrifugation [29,30]. Briefly, the homogenates were centrifuged at 2000× *g* for 5 min. The supernatant was centrifuged at 10,000× *g* for 15 min. The mitochondrial-enriched fraction (bottom) was resuspended in 2 mL of BSA-free isolation buffer and centrifuged at 10,000× *g* for 10 min. The pellet was resuspended in 200 µL of BSA-free isolation buffer and the mitochondrial total protein was measured by the Lowry method [31].

### 2.7. Mitochondrial H_2_O_2_ Production

Mitochondrial H_2_O_2_ levels were measured in a high-resolution respirometry O2k-Fluo Respirometer (Oroboros Instruments, Innsbruck, Austria) using Amplex red as a probe as previously described [32]. Isolated mitochondria were resuspended in 2 mL of MiR05 (110 mM sucrose, 20 mM HEPES, 20 mM taurine, 60 mM K-lactobionate, 3 mM MgCl_2_, 10 mM KH_2_PO_4_, 0.5 mM EDTA, and 1 g/L BSA (pH 7.1) plus 0.5 U/mL HRP. A calibration curve of H_2_O_2_ with known concentrations was employed to ensure the linearity of the assay, and sequential additions were employed to determine the production rate for each state. After the calibration curve, isolated mitochondria were loaded, and the rate of H_2_O_2_ production was measured (basal production). The rate of H_2_O_2_ production feeding CI (State 4 CI-Linked) was measured using 10 mM sodium pyruvate, 10 mM sodium malate, and 10 mM sodium glutamate (pyruvate–malate–glutamate, PMG). ADP was added to saturation (2.5 mM) to determine the rate of H_2_O_2_ production State 3 CI linked. Later, 10 mM sodium succinate was added to determine the rate of production in State 3 feeding by CI plus CII (State 3 CI + CII-Linked). Finally, the rate of H_2_O_2_ production by feeding β-oxidation was measured using 10 mM PC-C.

### 2.8. Glutathione Quantification

Total glutathione (GSH + GSSG) was evaluated by the enzymatic recycling method described by Rahman et al. [33], in which GSH reacts with DTNB forming TNB and glutathione-TNB adducts (GS-TNB). After, both GS-TNB and GSSG are reduced by GR to GSH, which in turn is oxidized by DTNB, forming TNB, which is detected at 412 nm. The amount of total glutathione calculated in this first step represents the sum of GSH and GSSG.

Next, GSSG was determined by the enzymatic recycling method mentioned above. Samples were previously treated with 0.2% 2-VP, which can covalently associate with GSH, leaving the oxidized form of glutathione as the only measurable substrate of the assay. GSH was calculated by subtracting GSSG from total glutathione (GSH + GSSG).

Briefly, the tumor extract was diluted with 120 μL of KPE buffer (0.1 μM potassium phosphate, 5 mM disodium EDTA, pH 7.5). Then, two separate samples of 20 μL each were used to measure either total glutathione or GSSG (these samples were previously treated with 0.2% 2-VP), mixed with DTNB (2.5 mM) and GR (250 U/mL). Finally, 0.24 mM NADPH was added, and the absorbance at λ = 412 nm was measured at intervals of 60 s, for 2 min. The rate of change in absorbance for each experiment was compared with GSH or GSSG standards.

### 2.9. Mitochondria Respirometry

The oxygen consumption experiments in the mitochondria of tumors were performed while using a high-resolution O2k FluoRespirometer (Oroboros Instruments, Innsbruck, Austria), as previously described [28]. Briefly, mitochondria were isolated from the tumor by differential centrifugation, as previously described [29,30]. Each experiment was carried out at 37 °C in 2 mL of mitochondrial respiration medium (MiR05) and initiated by the addition of the fresh mitochondria and the following conditions: the respiratory parameters were defined as (1). State 2 (S2) corresponds to the oxygen consumption in presence of mitochondria plus PMG (CI-linked substrates). (2). State 3 (S3) in which mitochondria oxygen consumption was stimulated by the addition of 2.5 mM ADP. (3). State 4 induced by 5 μM oligomycin (S4o). Respiratory control (RC) is defined as the S3/S4o ratio. OXPHOS-linked respiration (P) was calculated by the formula: S3–S4o. All the parameters were corrected by subtracting the non-mitochondrial respiration (ROX), which was obtained by adding 2 μM rotenone plus 11.25 μM diphenyleneiodonium chloride and 5 μM antimycin A and normalized by the concentration of protein of each mitochondrial sample.

### 2.10. Mitochondrial Membrane Potential (ΔΨm)

The changes in ΔΨm in the different respiratory states were measured as previously reported [32]. Briefly, the changes in safranin (S) O fluorescence (5 μM) in MiR05 medium were used to determine the ΔΨm. To stimulate CI-linked respiration, the respective substrates were added. Respiratory states were defined according to mitochondrial respiratory methodology in S3, which was obtained by the addition of 2.5 mM ADP, and in S4o, by the addition of 5 μM oligomycin. CCCP (5 μM) was added to completely dissipate the ΔΨm and to correct the non-specific interactions. Calibration curves of safranin were employed to ensure the linearity of the assay. Results were expressed as changes in measurable S O concentration (ΔμM S) in S3 or S4o with respect to CCCP uncoupling, and results were normalized per milligram protein and expressed as ΔμM S/mg protein.

### 2.11. Statistical Analysis

All experiments were performed in quintupled, and the data were analyzed as the mean ± standard error of the mean (SEM). *t*-Student test was used to determine the statistical significance of the experimental condition versus the control (SS group).

## 3. Results

### 3.1. GK-1 Decreases Catalase Enzyme Activity in TNBC

**Figure 2:**
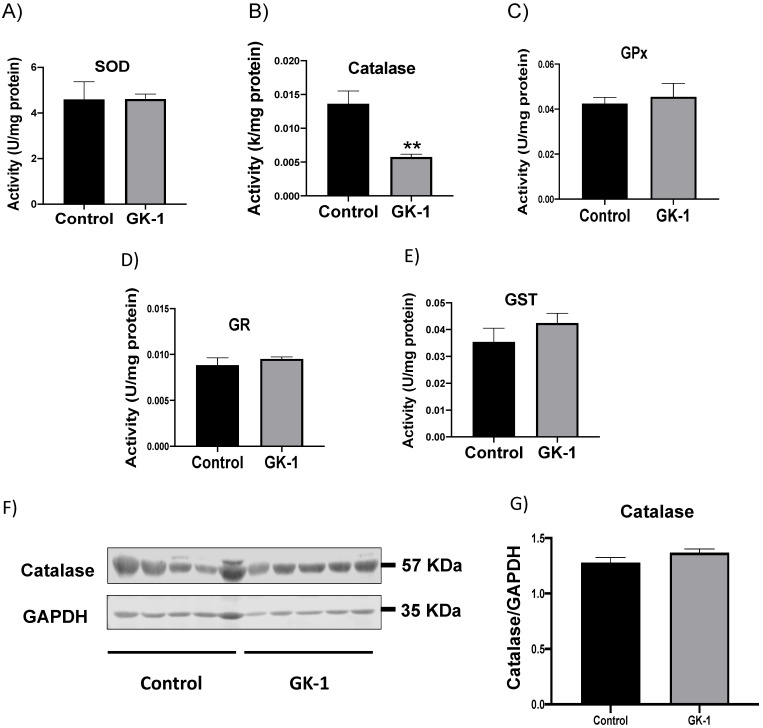
**GK-1 inhibits the enzymatic activity of catalase**. Enzymatic activity of (**A**) superoxide dismutase (SOD), (**B**) catalase, (**C**) glutathione peroxidase (GPx), (**D**) glutathione reductase (GR), (**E**) glutathione S-transferase (GST). (**F**) Representative immunoblotting and (**G**) densitometric analysis of catalase expression. Glyceraldehyde 3-phosphate dehydrogenase (GAPDH) was used as the loading control. *t*-Student test, control vs. GK-1, ** *p* < 0.005. Data are expressed as the mean ± SEM, n = 5.

Since one of the mechanisms related to the antitumor activity is the induction of OS, whether GK-1 can induce OS was evaluated. The activity of different key antioxidant enzymes associated with the prevention of OS, such as SOD, catalase, GPx, GR, and GST (Figure 2A–E) were evaluated. The catalase activity was found significantly reduced by GK-1 (Figure 2B). This finding is relevant since catalase is an enzyme that efficiently removes H_2_O_2_ produced during cell metabolism and thus prevents ROS production such as hydroxyl radical (^•^OH), which is produced during Fenton or Haber–Weiss reactions. Catalase protein levels were also measured to discern whether the decrease in its activity was due to the changes in its synthesis. However, we did not find changes in catalase protein levels (Figure 2F,G). Taken together, these results show that GK-1 induces the reduction of catalase function without affecting its expression, so GK-1 probably does not affect the transcription factors related to catalase expression. No effect of GK-1 was observed on the other antioxidant enzymes.

### 3.2. GK-1 Induces H_2_O_2_ Production in TNBC

**Figure 3:**
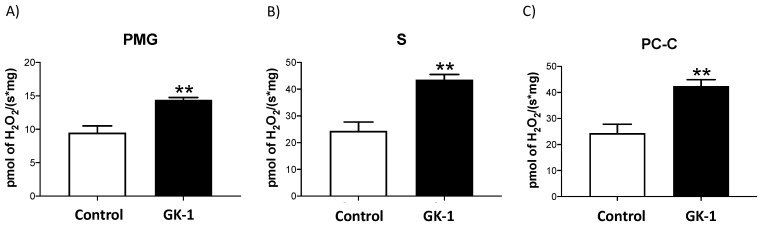
GK-1 induces hydrogen peroxide (H_2_O_2_) production in triple-negative breast tumors. (**A**) H_2_O_2_ production during the stimulation of oxidative phosphorylation (OXPHOS) through complex I (CI) with pyruvate–malate–glutamate (PMG). (**B**) H_2_O_2_ production during the stimulation of OXPHOS through complex II (CII) with sodium succinate. (**C**) H_2_O_2_ production during β-oxidation stimulation with palmitoyl-carnitine-CoA (PC-C). *t*-Student test, control vs. GK-1, ** *p* < 0.005. Results are expressed as mean ± SEM, n = 5.

Since GK-1 reduced catalase activity, suggesting that H_2_O_2_ degradation might be impaired. Indeed, H_2_O_2_ production of tumor mitochondria was found to be increased during OXPHOS stimulation. (Figure 3). We proved that feeding tumor cells with PMG, which are CI-linked substrates, induces mitochondrial H_2_O_2_ overproduction during GK-1 treatment in comparison with the control (Figure 3A). Furthermore, when we fed cells with CII-linked substrate (Figure 3B), we also observed higher rates of H_2_O_2_ production in tumor cells from GK-1-treated mice. Additionally, it was observed that PC-C (substrate for β-oxidation) also increased H_2_O_2_ production in GK-1-treated tumoral tissue cells (Figure 3C). Therefore, our results show that GK-1 induces overproduction of H_2_O_2,_ regardless of the type of substrate used, probably due to the reduction of catalase activity. However, it should be noted that ROS could also originate from mitochondrial metabolism due to mitochondrial dysfunction.

### 3.3. GK-1 Produces OS and Oxidative Damage in TNBC

**Figure 4:**
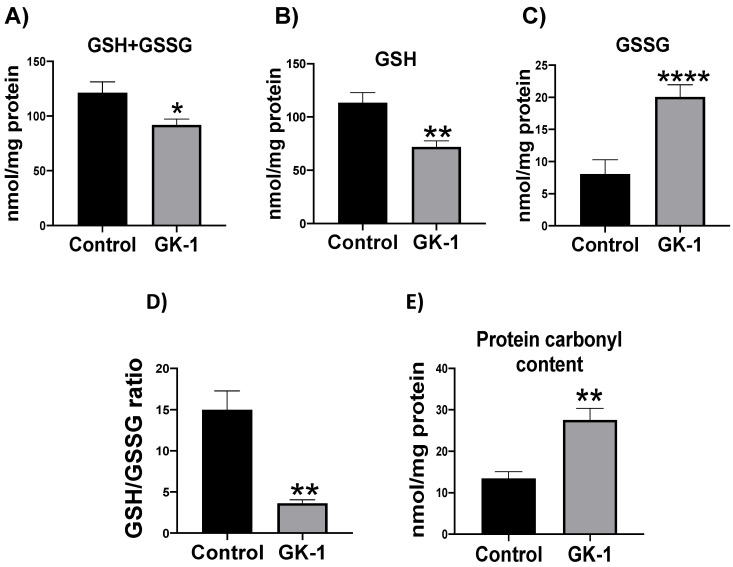
GK-1 stimulates OS and oxidative damage in TNBC. (**A**) Total glutathione (GSH+GSSG), (**B**) reduced glutathione (GSH), (**C**) glutathione disulfide (GSSG), (**D**) GSH/GSSG ratio, and (**E**) protein carbonyl levels in comparison with the control. *t*-Student test, control vs. GK-1, * *p* < 0.05, ** *p* < 0.005 and **** *p* < 0.00005. The results are expressed as the mean ± SEM, n = 5.

The high levels of H_2_O_2_ founded in GK-1-treated cells induce OS, can be represented by the reduced levels of GSH and the increased oxidized protein levels. Thus, we measured total glutathione (GSSG + GSH), GSH in its reduced and oxidized (GSSG) forms, the GSH/GSSG ratio, and oxidized protein levels (protein carbonyl groups). We found that GK-1 increased GSSG and reduced total glutathione and GSH in its reduced form, thereby decreasing the GSH/GSSG ratio (Figure 4A–D). We also observed that GK-1 significantly increases the protein carbonyl content (Figure 4E). Therefore, our results show that GK-1 promotes OS and oxidative damage.

### 3.4. GK-1 Decreases Mitochondrial Respiration in TNBC

**Figure 5:**
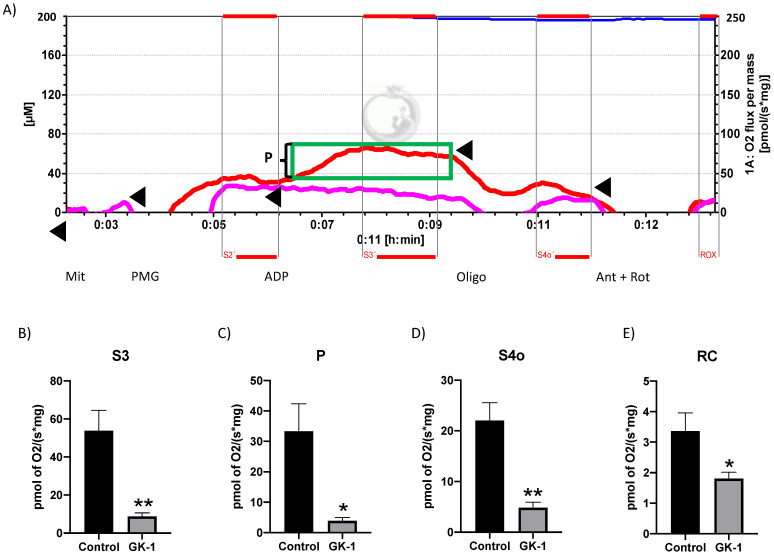
GK-1 inhibits cellular respiration and induces mitochondrial uncoupling in breast tumors. Mitochondrial respiratory parameters: (**A**) Representative tracings comparing experimental groups control vs. GK-1. We added the different substrates to the O2k FluoRespirometer chamber in the following sequence: mitochondria (Mit), pyruvate–malate–glutamate (PMG), adenosine phosphate (ADP), oligomycin (Oligo), and antimycin + rotenone (Ant + Rot). (**B**) ADP-stimulated respiration (state 3; S3), (**C**) Oxidative phosphorylation (OXPHOS)-linked-respiration (P), (**D**) State 4 was induced by 5 μM oligomycin (S4o), and (**E**) respiratory control (RC). State 2 (S2) corresponds to the oxygen consumption in presence of mitochondria plus PMG (CI-linked substrates). S3 corresponds to mitochondria oxygen consumption that was stimulated by the addition of 2.5 mM adenosine diphosphate (ADP). RC corresponds to the S3/S4o ratio. P was calculated by the formula: S3–S4o. All the parameters were corrected by subtracting the non-mitochondrial respiration (ROX). *t*-Student test, control vs. GK-1, * *p* < 0.05; ** *p* < 0.005. Data are expressed as mean ± SEM, n = 5. The red line indicates solution saline isotonic treatment (control), and the pink line indicates GK-1 treatment. The addition of the different substrates is indicated by triangles.

OS is a principal factor that induces mitochondrial dysfunction. We measured critical respiratory parameters to determine if OS and oxidative damage induced by GK-1 reduced mitochondrial bioenergetics. We observed that GK-1 reduced mitochondrial S3, P, S4o, and RC respiration (Figure 5A–E). Note that S3 corresponds to oxygen consumption in the presence of substrates that permit regular mitochondrial respiration of tumor treated with SS and treated with GK-1. S4o corresponds to cellular oxygen consumption that does not correspond to OXPHOS. P is linked to ATP production and RC, corresponding to the basal/leak respiration ratio. Thus, our results demonstrated that GK-1 reduces the overall mitochondrial respiration, thereby inducing mitochondrial dysfunction, which could be related to OS and oxidative damage induced by GK-1.

### 3.5. GK-1 Decreases The Levels of the ATP Synthase in TNBC

**Figure 6:**
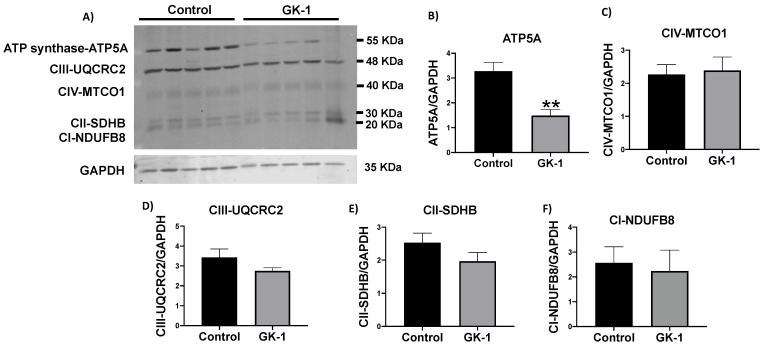
GK-1 decreases adenosine triphosphate (ATP) synthase. (**A**) Representative immunoblotting and (**B**) densitometric analysis of the levels of the ATP synthase-subunit (ATP5A), (**C**) cytochrome c oxidase subunit I (CIV-MTCO1), (**D**) ubiquinol–cytochrome c reductase core protein 2 (CIII-UQCRC2), (**E**) succinate dehydrogenase complex iron sulfur subunit B (CII-SDHB) and (**F**) reduced form of nicotinamide adenine dinucleotide ubiquinone oxidoreductase subunit B8 (CI-NDUFB8) in tumors treated with GK-1 and saline solution (control). Glyceraldehyde 3-phosphate dehydrogenase (GAPDH) was used as the loading control. *t*-Student test, control vs. GK-1, ** *p* < 0.005. Data are expressed as mean ± SEM, n = 5.

Since we observed a decrease in mitochondrial respiration with GK-1 treatment, we evaluated whether this deficiency was due to a decrease of mitochondrial complexes and ATP synthase. Thus, we measured the subunits levels of NDUFB8 for CI, SDHB for CII, UQCRC2 for CIII, MTCO1 for CIV, and ATP 5A for ATP synthase by immunoblot (Figure 6). Although we did not find a decrease in the levels of mitochondrial complexes, we observed that GK-1 significantly decreased the ATP5A subunit of ATP synthase (Figure 6B). This result suggests that decreased P respiratory parameter can be partially attributable to lower ATP synthase subunit levels.

### 3.6. GK-1 Decreases VDAC1 in TNBC

**Figure 7:**
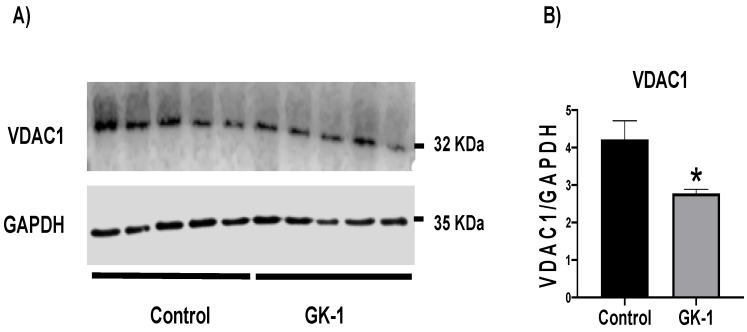
GK-1 decreases voltage-dependent anion channel 1 (VDAC1) expression. GK-1 decreases VDAC1 in triple-negative breast tumor. (**A**) Representative immunoblot and (**B**) densitometric analysis of mitochondrial mass marker VDAC1. Glyceraldehyde 3-phosphate dehydrogenase (GAPDH) was used as the loading control. t-student test, control vs GK-1, * *p* < 0.05. Data are expressed as mean ± SEM, n = 5.

VDAC1, as a gatekeeper of the mitochondria, is essential for cell death and survival signaling pathways. It is involved in the coupling of cellular energy demand to mitochondrial ATP production, as this transporter is the main conduit for the transport of metabolites across the outer mitochondrial membrane (OMM). Moreover, VDAC1 is considered as a marker of mitochondrial because it is an essential transport, and its levels are related to the function and mass of mitochondria [34]. Thus, we measured VDAC1 levels during GK-1 treatment, founding that GK-1 significantly reduced VDAC1 levels (Figure 7). Although we need other markers of mitochondrial mass to conclude that GK-1 downregulates mitochondrial mass such as adenine nucleotide translocator (ANT), our result suggests that GK-1 could modulate mitochondrial mass processes such as respiration and that this effect on transporters such as VDAC1 is part of its function as an antitumoral molecule.

### 3.7. GK-1 Decreases the Mitochondrial Membrane Potential (ΔΨm) of TNBC

**Figure 8:**
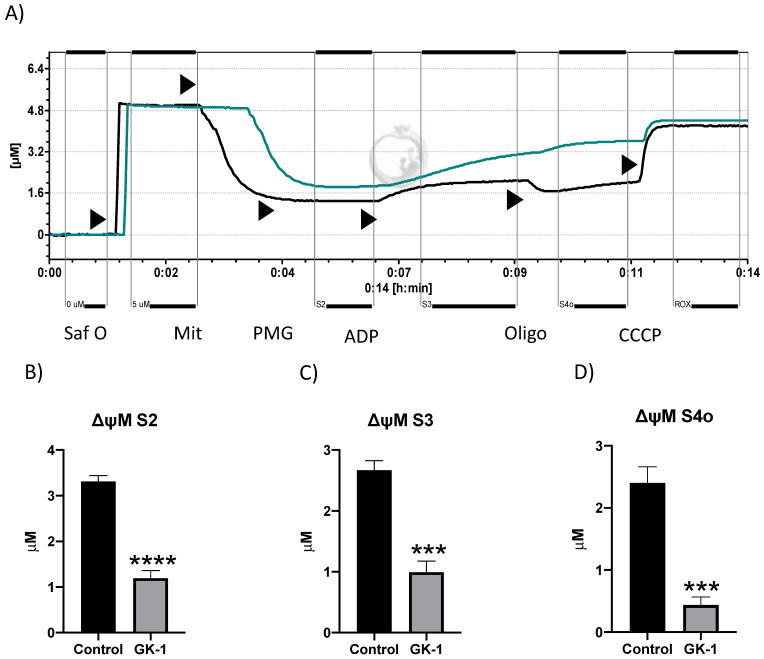
GK-1 dissipates mitochondrial membrane potential (ΔΨm) in mouse breast tumors. (**A**) Representative tracings of mitochondrial membrane potential evaluation using complex I (CI)-associated substrates comparing experimental groups control vs. GK-1. We added the different substrates to the O2k FluoRespirometer chamber in the following sequence: Safranine (Saf O), mitochondria (Mit), pyruvate–malate–glutamate (PMG), adenosine phosphate (ADP), oligomycin (Oligo), and carbonyl cyanide 3-chlorophenylhydrazone (CCCP). (**B**) Membrane potential in state 2 (S2), (**C**) membrane potential associated in state 3 (S3), and (**D**) membrane potential in state 4 induced by oligomycin (S4o). The potential in state 2 is obtained with the addition of the sample plus the substrates. *t*-student test, control vs GK-1, *** *p* < 0.0005 and **** *p* < 0.00005. Data are expressed as mean ± SEM, n = 5. The green line indicates solution saline isotonic treatment (control), and the black line indicates GK-1 treatment. The addition of the different substrates is indicated by triangles.

Since the decrease in the VDAC1 and OXPHOS capacity can be related to changes in ΔΨm, we measured it in different respiratory states: when mitochondria are producing ATP (S2 and S3), and in non-ATP producing state 4 induced by oligomycin (S4o). We found that GK-1 decreased ΔΨm in all mentioned above states (Figure 8A–D). In conclusion, our results show that GK-1 induces a failure of the ΔΨm in both ATP-producing and non-ATP-producing respiratory states, which is closely related to mitochondrial uncoupling and the reduction of tumor growth.

### 3.8. GK-1 Stops Autophagy Flux in TNBC

**Figure 9:**
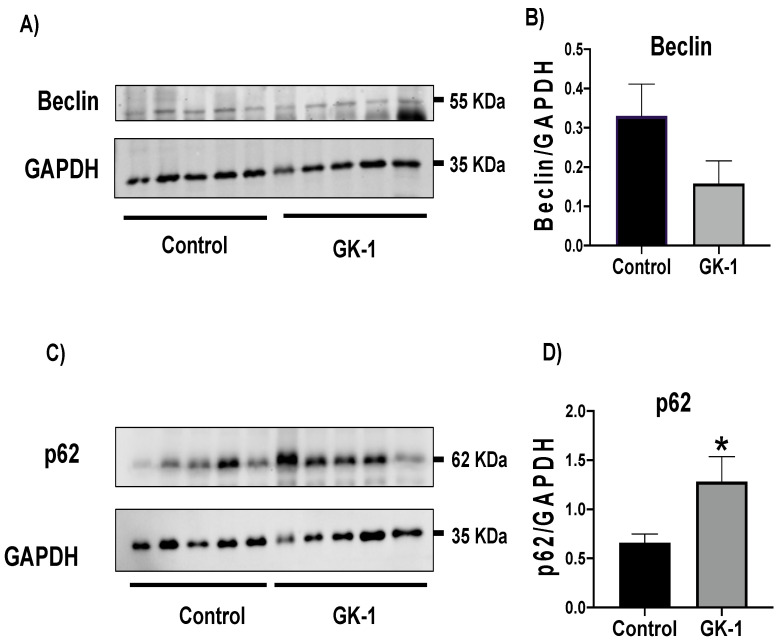
GK-1 disrupts autophagy flux. (**A**,**C**) Immunoblots and (**B**,**D**) densitometric analyses of Beclin and p62, respectively, in tumors treated with GK-1 and saline solution (control). Glyceraldehyde 3-phosphate dehydrogenase (GAPDH) was used as the loading control. *t*-Student test, control vs. GK-1, * *p* < 0.05. Data are expressed as mean ± SEM, n = 5.

Due to the increase in ROS and OS, we wondered if the damage caused by OS, demonstrated by an increase in protein carbonyl content (Figure 4E), activates autophagy to remove altered proteins and damaged organelles. It has been shown that when p62 binds to autophagic substrates and delivers them to autophagosomes for degradation, p62 itself is degraded, and p62 is depleted during autophagic flux. Conversely, when the autophagic flux is stopped, a corresponding increase in p62 levels is observed because p62 is not degraded within the autophagosome. Thus, when autophagy flux is inhibited, p62 accumulates in the cell [35]. We measured Beclin and p62 as autophagy markers, finding that GK-1 induced a tendency to decrease Beclin and significantly increases p62 (Figure 9). Both results suggest that GK-1 could induce a disruption in the autophagic flux necessary to restore damaged proteins and organelles such as mitochondria.

## 4. Discussion

**Figure 10:**
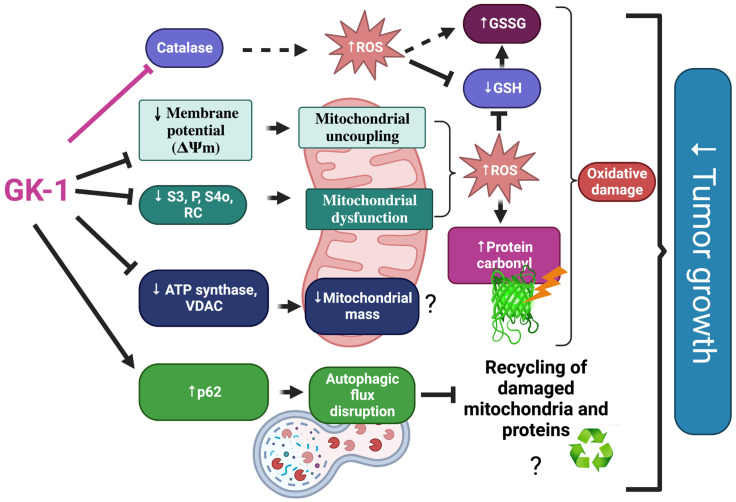
Integrative schema. GK-1 decreases catalase activity and mitochondrial respiration evaluated by state 3 (S3), oxidative phosphorylation-linked respiration (P), state 4 induced by oligomycin (S4o) and respiratory control (RC) and mitochondrial membrane potential (ΔΨm) inducing mitochondrial dysfunction and mitochondrial uncoupling. These alterations could induce reactive oxygen species (ROS) overproduction, oxidative stress, and oxidative damage (protein carbonyl formation, decrease in glutathione (GSH) levels and increase in glutathione disulfide (GSSG) levels). Moreover, GK-1 decreases adenosine triphosphate (ATP) synthase and voltage-dependent anion channel 1 (VDAC1) levels affecting mitochondrial mass and decreases Beclin and increases p62 that are associated with disruption of autophagic flow, reducing triple-negative breast tumor growth. The figure was created with BioRender.com.

In different models of breast cancer and melanoma, it has been observed that GK-1 has an antitumor effect decreasing angiogenesis, metastasis, as well as tumor growth, which has been related to the modulation of different factors such as the decrease of different cytokines associated with malignancy such as basic fibroblast growth factor (bFGF), granulocyte macrophage-colony stimulating factor (GM-CSF), tumor necrosis factor alpha (TNF-α) or chemokine (C-X-C motif) ligand 9 (CXCL9), increasing interleukin (IL)-6 and IL-12 levels [5]. It has also been found that GK-1 induces anti-tumoral cluster of differentiation 8 (CD8) T cell response associated with the downregulation of the programmed cell death protein 1 (PD-1)/ programmed death-ligand 1 (PD-L1) pathway [10]. The increase of ROS and OS are conditions associated with mitochondrial damage and dysfunction that have been linked to cancer development and its elimination [36]. Considering this, we wondered if GK-1 targets the redox state, mitochondrial bioenergetics, and autophagy process. We found that in the TNBC mice model, GK-1 induces OS and oxidative damage since an increased mitochondrial H_2_O_2_ production, GSSG, and protein carbonyl contents, and a reduction of the antioxidant activity of catalase, GSH content, and the GSH/GSSG ratio (Figure 2, Figure 3 and Figure 4) was found. This prooxidant effect of GK-1 is similar to that caused by peptides from the venom of the marine cone snail *Conus vexillum*, which induces OS by increasing ROS and reactive nitrogen intermediates as well as decreasing the activities of antioxidant enzymes catalase and SOD, causing oxidative damage such as lipid and protein oxidation in carcinoma cells. This induction of OS and its cytotoxic effects confer to venom-anticancer properties [37]. GSH is the main antioxidant in the mitochondria and helps to maintain mitochondrial homeostasis, so when a decrease in GSH content and H_2_O_2_ production increase occurs, OS is promoted, which induces mitochondrial oxidative damage and a decrease in cell viability [38]. Thus, the OS induced by GK-1 would compromise tumor growth and metastasis as a consequence of the oxidative damage that occurs in the mitochondria. We found that tumors treated with GK-1 peptide decreased their respiratory S3, S4o, P, and RC parameters (Figure 5B–E). The S3 respiration corresponds to the oxygen consumption in the presence of all the substrates that allow the mitochondria to perform OXPHOS, and we found that this decreases significantly with GK-1. The S4o refers to cellular oxygen consumption that does not correspond to OXPHOS, which is also decreased by GK-1 treatment. Moreover, P, linked to ATP production and RC, also decreases, showing that mitochondria cannot use adequate oxygen to produce ATP. It should be noted that if the RC index is low, it indicates that mitochondria proton leak processes increased, and the oxygen is being used for other processes not associated with the production of ATP. For instance, one of these oxygen processes could be used by ROS production [39], which agrees with the higher levels of mitochondrial H_2_O_2_ production. These results suggest that GK-1 induces mitochondrial dysfunction, where mitochondria do not use oxygen efficiently for ATP production. Moreover, we observed that tumors treated with GK-1 have fewer ATP synthase subunit levels, which compromises tumor cell respiration, demonstrated by the significant decrease in S3, P, and RC (Figure 5B–D). The cell growth is dependent on mitochondria since this organelle provides not only energy in ATP form but metabolites that induce cell growth [40]. Regarding the latter, we found that ATP synthase and VDAC1 levels are decreased in GK-1-treated tumor cells (Figure 7), which agrees with the reduction of tumor, as ATP synthase and VDAC1 are essential proteins in mitochondrial metabolism and ROS-mediated cell death [34,41]. On the other hand, the results of ΔΨm analysis show that GK-1 decreases it in S2, S3, and S4o, indicating that the mitochondria are not capable to maintain the polarization status. In the case of S3, the ΔΨm loss explains the decreased ATP production capacity in mouse TNBC. The loss of ΔΨm is closely associated with cell viability since a decrease in the potential can induce cell death [42]. These effects agree with the effect of CIGB-552, a synthetic anti-tumor peptide developed by The Center for Genetic Engineering and Biotechnology (CIGB), which decreases SOD1 activity, reduces cellular antioxidant capacity, and promotes ROS production. The latter induces more damage to mitochondria and increases the oxidative damage of lipids and proteins in tumor cells, leading to apoptosis-mediated cell death. In this study, authors show that CIGB-552 effect reduces tumor growth of human colon tumor xenografts [43]. One of the processes that allow the elimination of proteins and organelles damaged by OS is autophagy, so by measuring the markers of this process, we can assume that tumor cells could be protecting themselves from OS damage. However, we found that GK-1 increases p62 autophagy marker, indicating that GK-1 stops autophagic flux (Figure 9). Yan et al. showed that invading human primary non-small cell lung cancer (NSCLC) exhibits higher autophagic flux than cancer cells inside the tumor body; when this autophagy flux is stopped, tumor invading capacity decreases [44]. It is important to note that we only measured p62 as a marker of autophagic flux. Measuring LC3 turnover assay together with electron microscopy techniques will define whether GK-1 reduces autophagic flux or not. Moreover, the measure of additional mitochondrial mass markers such as mitochondrial DNA or imaging mitochondrial network by electron microscopy would help to overcome these limitations of our work. Even with these limitations, our results make an approximation of the effect of GK-1 on autophagic flux and mitochondrial mass, which suggest that the arrest of autophagic flow by GK-1 could decrease the invasion and metastasis capacity of 4T1 cells, as has been shown by Torres-García et al. [5]. However, more investigation is necessary to conclude these effects. Thus, we found that GK-1 induces OS, mitochondrial dysfunction characterized by ΔΨm loss, uncoupling, and higher ROS production, as well as a probable disruption of autophagic flow, which could compromise tumor cell viability. It is also probable that GK-1 directly affects mitochondrial homeostasis, inducing mitochondrial dysfunction, which in turn would promote ROS production and OS, having positive feedback in ROS generation, which is reinforced by the decreased catalase activity during GK-1 treatment. The latter, together with the stop of autophagy flux induced by GK-1 will reduce tumor growth and metastasis (Figure 10) [5]. Notably, it is important to highlight that in preclinical studies, GK-1 peptide was safe as the absence of subchronic toxicity and mutagenicity was found [45], accounting for these effects only in tumoral tissues. Discovering the exact mechanism of how GK-1 induces mitochondrial dysfunction deserves further investigation because this could open up new avenues for applying drugs that exert tumor elimination.

## 5. Conclusions

GK-1 induces OS and oxidative damage, which is associated with mitochondrial uncoupling and higher H_2_O_2_ production by this organelle. This induces lower efficiency in mitochondrial ATP production in mouse TNBC induced by 4T1 cells. In addition, we show that GK-1 induces alterations in autophagic flow. Consequently, these GK-1-treated tumors are not in optimal states of recycling mitochondria and damaged proteins, which might be associated with its capacity to control tumor growth.

## Data Availability

Data are contained within the article.

## Abbreviations

ΔΨm	Mitochondrial membrane potential
2-VP	2-vinylpyridine
ADP	Adenosine diphosphate
ANT	Adenine nucleotide translocator
ATP	Adenosine triphosphate
ATP5A	ATP synthase subunit
BSA	Bovine serum albumin
BC	Breast cancer
bFGF	Basic fibroblast growth factor
CCCP	Carbonyl cyanide 3-chlorophenylhydrazone
CD8	Cluster of differentiation 8
CICUAL	Comité Interno para el Cuidado y Uso de los Animales de Laboratorio
CDNB	1-Chloro-2,4-Dinitrobenzene Solution
CI	Complex I
CII	Complex II
CI-NDUFB8	CI-NADH: ubiquinone oxidoreductase subunit B8
CII-SDHB	CII-Succinate dehydrogenase B
CIII-UQCRC2	CIII-Ubiquinol-cytochrome c reductase core protein 2
CIV-MTCO1	CIV-Cytochrome c oxidase I
CXCL9	Chemokine (C-X-C motif) ligand 9
DNPH	2,4-dinitrophenylhydrazine
DPI	Diphenylene iodonium
DTNB	5,5-dithio-bis (2 nitrobenzoic) acid
EDTA	Ethylenediaminetetraacetic acid
ER	Estrogen receptor
ETS	Electron transport system
GAPDH	Glyceraldehyde 3-phosphate dehydrogenase
GM-CSF	Granulocyte macrophage-colony stimulating factor
GSH	Reduced glutathione
GSSG	Glutathione disulfide, glutathione oxidized
GSSG + GSH	Total glutathione
GPx	Glutathione peroxidase
GR	Glutathione reductase
GST	Glutathione S-transferase
GSH-TNB	Glutathione-TNB adducts
HEPES	4-(2-hydroxyethyl)-1-piperazineethanesulfonic acid
HER2	Human epidermal growth factor receptor 2
HRP	Horseradish peroxidase
H_2_O_2_	Hydrogen peroxide
IL	Interleukin
KH_2_PO_4_	Potassium phosphate monobasic
MgCl_2_	Magnesium chloride
mROS	Mitochondrial ROS
MCP-1	Monocyte chemoattractant protein 1
MIP-1α	Macrophage inflammatory protein-1 alpha
MiR05	Mitochondrial respiration medium
MnSOD	Manganese superoxide dismutase
NAC	N-acetyl-l-cysteine
NaCl	Sodium chloride
NADH	Nicotinamide adenine dinucleotide
NADP	Nicotinamide adenine dinucleotide phosphate
NaF	Sodium fluoride
NaH_2_PO_4_	Sodium phosphate monobasic
Na_2_HPO_4_	Sodium phosphate dibasic
NaN_3_	Sodium azide
Na_3_VO_4_	Sodium orthovanadate
NBT	Nitroblue tetrazolium
NIH	National Institute of Health
NSCLC	Non-small cell lung cancer
OD	Optical density
^•^OH	Hydroxyl radical
OMM	Outer mitochondrial membrane
OS	Oxidative stress
OXPHOS	Oxidative phosphorylation
P	OXPHOS-linked respiration
PD-1	Programmed cell death protein 1
PD-L1	Programmed death-ligand 1
PC-C	Palmitoyl-carnitine-CoA
PMG	Pyruvate plus malate and glutamate
PMSF	Phenylmethylsulfonyl fluoride
PR	Progesterone receptor
PVDF	Polyvinylidene fluoride membranes
RC	Respiratory control
RIPA	Radioimmunoprecipitation assay
ROS	Reactive oxygen species
ROX	Non-mitochondrial respiration
S2	State 2
S3	State 3
S4o	State 4 induced by oligomycin
S	Safranin
SEM	Standard error of the mean
SS	Saline solution
SDS	Sodium dodecyl sulfate
SOD	Superoxide dismutase
TBST	Tween-tris buffered solution
TMPD	Tetramethyl-p-phenylene diamine
TNB	5-thio-2-nitrobenzoic acid
TNBC	Triple-negative breast cancer
TNF-α	Tumor necrosis factor alpha
U	Units
UV	Ultraviolet
VDAC1	Voltage-dependent anion-selective channel 1
